# Towards Better Governance on Biosafety and Biosecurity: China’s Advances and Perspectives in Medical Biotechnology Legislation

**DOI:** 10.3389/fbioe.2022.939295

**Published:** 2022-07-04

**Authors:** Yang Xue, Lijun Shang

**Affiliations:** ^1^ Law School, Tianjin University, Tianjin, China; ^2^ Center for Biosafety Research and Strategy, Tianjin University, Tianjin, China; ^3^ School of Human Sciences, London Metropolitan University, London, United Kingdom; ^4^ Biological Security Center, London Metropolitan University, London, United Kingdom

**Keywords:** biosafety, biosecurity, medical biotechnology legislation, China, precautionary principle

## Abstract

In this paper, we systematically investigated and assessed China’s evolving medical biotechnology legislative and regulatory regime. 89 laws, rules, measures, guidelines, and views from 1985 to 2022 were systematically analogized and 28 were found to be involved in medical biotechnology legislation, including the recently passed Biosecurity Law. We classified the legislations and performed a comparative analysis for their legal binding based on the legal subject and extent of application, then further analyzes some of the legislative challenges in governing medical biotechnology risks in the context of China’s upgrading its regulatory and legal regime in the last 3 years. We concluded that policymakers in China have now incorporated medical biotechnology-related biosafety and biosecurity into the national strategic goals of a “People-Centered” approach to establish and foster an ecological civilization, particularly in the aftermath of the “He Jiankui affair.” Instead of relying on a patchwork of existing regulations and measures relating to the emerging field of medical biotechnology, China is attempting to integrate a patchwork of existing regulations and measures into a comprehensive legal framework, such as the constitution, National Security Law, Biosecurity Law, administrative regulations, departmental and local rules, and has begun to use the Civil Code and Criminal Law to explicitly identify actions relating to medical biotechnology. In general, China follows the “precautionary principle” as it thinks that uncertainty in science and technology should not be used to justify delaying the adoption of measures to prevent injuries or dangers, stating that whoever advances biotechnology must face the burden of proof of no harm. There would be a need to impose oversight for prevention and precaution if any biotechnology breakthrough that carries risks on which no scientific consensus has been reached. We argued that the “top-down” formulation of general objectives by the active political leadership and “bottom-up” innovation in the implementation are the keys to achieving these goals. Given the rapid advancements in medical biotechnology, countries all over the world must examine the governance landscape around biosafety and biosecurity and quickly consider options for their own comprehensive, credible, and long-lasting regulatory frameworks and experiences learned from China’s governance will help chart a scalable future roadmap.

## Introduction

Life sciences and biotechnologies contribute to a better knowledge of diseases as well as the creation of novel medications, vaccines, innovative therapies, and medical devices through research and applications. Their rapid scientific and technological advancements, on the other hand, could create several risks, particularly to the safety and security of health-related research. For example, although genome editing has the potential to fix harmful genes such as spinal muscular dystrophy, thalassemia, and retinal macular degeneration (Rees and Liu, 2018; Frangoul et al., 2021), off-target consequences could potentially worsen the disease or result in death (Watters, 2018). Even though biotechnology has shown great industrial development potential in agriculture, forestry, energy, environmental protection, and materials, to implement the “People-Centered” development philosophy, China has focused the primary task of biotechnology application on improving people’s health guarantee ability, that is, focusing on medical biotechnology such as drugs, vaccines, advanced diagnosis and treatment technology (National Department and Reform Commission of the People’s Republic of China, 2022; China Daily News, 2022a). As a result, China’s biotechnology legislation focuses on medical biotechnology regulation, such as human genetic engineering, clinical research, and ethics, as well as export control of dual-use goods and technologies and pathogenic microorganisms oversight. Since the 1980s, China has had “administrative regulations” to address these challenges, but the legal basis for punishment for those rule-breakings has lagged. The aforementioned “administrative regulations” are based on measures or principles that are less enforceable than the laws that have been put into use in China in the last 3 years. In particular, due to the lack of essential punishment effectiveness in these measures, the legal basis is lacking to assume accountability for technological damage compensation and dispute resolution procedures when dealing with occurrences like the “He Jiankui affair” (Krimsky, 2019). China has been working constantly to improve its medical biotechnology regulatory and legal systems, recognizing that developing governance policies need to catch up with the rapid progress of science and technologies, as accidents involving biosafety and biosecurity are still inevitable. During this process, several important and urgent questions need to be answered, such as: what is the most efficient method to prepare for prohibitive medical biotechnology risk policies? Is it possible and effective to use the precautionary principle to impose oversight for preventive and precautionary measures? Should the relevant rules be based on traditional biotechnology applications or should they be enforced by measures, guidelines, or legislation? Meanwhile, there are developing challenges in managing the risks of medical biotechnology in China that also deserve special attention: 1) a predicament in maintaining a delicate balance between the regulations’ specificity and excessive generality; 2) a dilemma between the demand for cooperation and the distribution of authority and responsibility among multiple government departments and agencies; 3) a shortage of nontraditional scientists’ provisions; 4) a lack of effective regulatory restraint on emerging biotechnologies; and 5) consequences of the relative regulations resulting in insufficient efforts in biologists’ biosecurity awareness and moral self-discipline, particularly in terms of execution.

In this paper, we provided a systematic analysis of the medical biotechnology legal regime in China. We first explain and appraise China’s legislative progress in medical biotechnology legislation before the “He Jiankui affair,” as well as the context in which China has attempted to strengthen its biotechnology legislation. 89 laws, rules, measures, guidelines, and views from 1985 to 2022 were systematically analogized and found 28 were found to be involved in medical biotechnology legislation, including the recently passed Biosecurity Law. It also classifieds the legislations and performed a comparative analysis for their legal binding based on the legal subject and extent of application, then further analyzes some of the legislative challenges in governing medical biotechnology risks in the context of China’s upgrading its regulatory and legal regime in the last 3 years. We then conducted a preliminary examination of China’s medical biotechnology legal framework, which is based on the Biosecurity Law. The review of China’s medical biotechnology legislative and regulatory systems up to the passing of the Biosecurity Law in 2021 is then followed by scrutiny of the most recent developments in the country’s medical biotechnology legislation. We finally concluded with the prospect of China’s medical biotechnology legislation and governance. We would hope that the lessons and experiences from China will provide useful information for other countries around the world.

## The Progressive Legislation and Legal Governance on Medical Biotechnology in the First Round in China

The legislation on medical biotechnology in China can be roughly divided into two main stages. The first round of important work began in the 1980s. In 1985 and 1986, the Measures on the Preservation of Medical Microorganisms and the Regulations on the Administration of Deposit of Microorganisms were introduced by the Ministry of Health (MoH), which represents the start of China’s advances in medical biotechnology legislation. The legislation only includes a section on the preservation and deposit of microorganisms due to the then undeveloped medical biotechnologies in China. The Measures on the Management of Genetic Engineering Safety, released by the State Science and Technology Commission in 1993, are one of the most important pieces of law. This regulation established risk classification systems for genetic engineering activities with a critical assessment as well as biosafety surveillance within the continuum of the “research-experimental-application” in the approval process as a standard that guides researchers, reviewers, and regulators (China National Center for Biotechnology Development, 2019). In general, China promulgated its governance system and focused on several fronts such as pathogenic microorganisms, clinical research, genetic engineering, and modification, including some measures and/or professional guidance or ethical principles versus medical biotechnology risks concerning biosafety and biosecurity since the 1990s. China has established a regulatory and legislative framework for medical biotechnology in response to international and domestic scenarios. The key motivations can be summarized below.

First, zoonotic diseases such as severe acute respiratory syndrome (SARS), Ebola, Middle East respiratory syndrome (MERS), West Nile fever, and Rift Valley fever, as well as COVID-19, have been on the rise all over the world in the last two decades (Bouskill and Smith, 2019; Zhang et al., 2022). These pandemics have clearly illustrated the significance of using new tools and methods to combat deadly diseases, as well as the potential biosafety concerns, both of which are critical in establishing a consensus among China’s regulators on a more proactive approach to medical biotechnology risks. The SARS outbreak and a SARS lab leak from the National Center for Disease Control and Prevention (CDC) have also accelerated the establishment and improvement of the regulatory structure that allows scientists to handle dangerous diseases and unpredictable experiments with care (Normile, 2004). As a result, in the years after 2003, China enacted a flurry of medical biotechnology-related regulations. The Regulations on the Management of Pathogenic Microorganisms Laboratories Biosafety (2004), as well as the List of Human Pathogenic Microorganisms (2006), are two of the most essential pieces of legislation. The former clearly defined the pathogenic microorganism classification scheme and biology laboratory hierarchical management, whereas the latter defined the biosafety level required (BSL-1,2,3,4) for experimental activities such as virus cell culture, experimental animal infection, non-cultured infectious material operation, and inactivated materials operation (China National Center for Biotechnology Development, 2019). Crucially, China will have established the principles of biotechnology classification and hierarchical administration by then.

Second, related regulations and lawmaking are also being considered in other countries, and China is keeping up with the world. For example, in America, the government’s initial efforts to provide oversight of genetic engineering activities arose in the mid-1970s (Wilson, 1993), and it established the world’s first document on the governance of biotechnology, entitled the Guidelines for Research Involving Recombinant DNA Molecules in 1976. In 2003, the National Research Council under the US National Academy of Sciences, Engineering, and Medicine (NASEM) published Biotechnology Research in an Age of Terrorism, as the first effort to incorporate biotechnology governance into the system of national security (National Research Council, 2004). Since 2010, the USA has accelerated to issue a series of policies to strengthen its biotechnology governance, including the Policy for Oversight of Life Sciences Dual Use Research of Concern (2012), Biosafety and Biosecurity (2014), Policy for Institutional Oversight of Life Sciences Dual Use Research of Concern (2014), National Strategy for Modernizing the Regulatory System for Biotechnology Products (2016), Recommended Policy Guidance for Departmental Development Review Mechanisms for Potential Pandemic Pathogen Care and Oversight (P3CO) (2017), (Husbands, 2018), and the National Biodefense Strategy released under the Trump administrations in 2018. In general, in the United States, oversight frameworks already exist for many activities of modern biological science, including research involving humans, animals, microorganisms and toxins, and recombinant DNA. Oversight also occurs regarding laboratory worker safety, use of federal funds in research, and transport and containment of dangerous agents. Oversight is frequently, but not exclusively, tied to public funding or the need to gain regulatory approval to market or distribute a product. In summary, the USA takes governance through three channels—legal statutes and regulations; guidelines and guidelines set up by funding agencies; and voluntary policy implementation of unregulated science or entities (Berger et al., 2018). China has drawn on the experience of other countries’ developments, which has given it reasons to take swift and necessary actions. But China also carefully monitors its national circumstances, which will be discussed in the later sections.

Third, as evidenced by the advancement of the most relevant international convention—the Biological and Toxin Weapons Convention (BTWC)—international attention has gradually shifted to its focus on biotechnology governance. The BTWC, as the only instrument entered into force by the United Nations General Assembly with legally binding relevance in the field of international biosecurity governance, has gradually formed a prevention network universally recognized by all state parties to eliminate the threat of biological weapons and prevent their spread. Furthermore, UNSCR 1540, which was adopted by the United Nations in 2004, requires States Parties to take and enforce effective measures to establish domestic controls to prevent the proliferation of nuclear, chemical, and biological weapons and their delivery systems, including appropriate controls over related materials. To that end, China has implemented relevant export control regulations for dual-use goods and technologies, such as the Regulations of the People’s Republic of China on Export Control of Biological Dual-Use Items and Related Equipment and Technologies (China National Center for Biotechnology Development, 2019). BTWC has addressed biotechnology since its entering into force in 1975. However, about 20 years ago, the first meeting of experts took place that, in parts at least, has focused on discussing risks to the convention arising from modern biotechnology (Chyba, 2006). For example, the BTWC established the Meetings of Experts (MXs) series, which included MX2. The overarching theme for MX2 is “Review of Developments in the Field of Science and Technology Related to the Convention, including the enhanced implementation of the identification of potential benefits and risks of new science and technology developments relevant to the Convention, with special attention to positive implications” (Biological Weapons Convention, 2020). Since the BTWC’s 5th Review Conference of State Parties in 2002, the convention has focused more on discussing the establishment and implementation of preventing the misuse of bioscience and biotechnology research that may be used for prohibited purposes. Meanwhile, all other scientific and technological developments relevant to the Convention, as well as the activities of relevant multilateral organizations such as the WHO, OIE, FAO, IPPC, and OPCW, prompted China to submit working papers on the “Proposal for the Development of a Model Code of Conduct for Biological Scientists” (BWC/CONF.VIII/WP.30) to improve global biosafety management for the Eighth BWC Review Conference in 2016 (BWC/CONF.VIII/WP.30, 2016). In this recommendation, China urges biological researchers to assess the risks of their investigations and take practical efforts to avoid or cancel any potentially hazardous initiatives.

Fourth, China has been a pioneer in the development and commercialization of genetically modified organisms (GMOs), for example (Cao, 2021), and this prioritizes biomedical research, especially in the field of CRISPR gene editing (Shen, 2014; Cyranoski, 2016). This inevitably requires China to strike a balance between growing medical biotechnology and adhering to worldwide research norms in bioethics as well as controlling biosafety and biosecurity, especially after the “He Jiankui affair” and several other types of research (Liang et al., 2015; Ma et al., 2017; He, 2022). Such gene-editing affairs served as more than a wake-up call, highlighting the inadequacies of China’s current biosafety regulatory regime while also posing significant challenges to the country’s biosafety governance. For example, in the field of governance on human germline editing, the Ministry of Health introduced the Quality Control Points for Clinical Researches of Somatic Human Genome Therapy as early as 1993. As the technology at that time was far from being able to genetically edit human germline cells, the legislation did not yet include a section on this aspect. Since 2001, the Ministry of Health of China issued the Measure for Human Assisted Reproductive Technology (2001), Specifications for Human Assisted Reproduction Technology (2003), and Ethical Principles for Human Assisted Reproductive Technology and Human Sperm Bank (2003) in quick succession, which focused on *In Vitro* Fertilization (IVF) and chimeric embryo technology yet did not regulate genetic modifications with the special article. In 2003, the Ethical Guidelines on Human Embryonic Stem Cell Research were issued by the Ministry of Science and Technology and the Ministry of Health, which were only for stem cells and can also be extended to regulate germline cells. The term of *in vitro* culture for blastocysts acquired through *in vitro* fertilization (IVF), somatic cell nuclear transfer, monoclonal methods, or genetic changes shall not exceed 14 days from the time of fertilization or nuclear transfer, according to Article 6. Human blastocysts obtained for research purposes in the preceding paragraph shall not be implanted into the reproductive systems of humans or any other animals (China National Center for Biotechnology Development, 2019). In 2016, the Ministry of Medical and Health of China issued the Measures for Ethical Review of Biomedical Research Involving Human Beings, which clearly defined that each medical and health institution should establish its own ethics committees with the following mandates: 1) informed consent; 2) risk controllability; 3) privacy protection; 4) financial assistance; 5) compensation by the law; and 6) special protection for subjects in special groups such as children, pregnant women, mentally retarded, and people with mental disorders (Ministry of Medical and Health of the People’s Republic of China, 2016). In 2017, the Ministry of Science and Technology of China issued the Measures for the Safety Management of Biotechnology Research and Development as an administrative regulation governing emerging biotechnologies, under which state authorities will make determinations about the (im)permissibility of human germline or heritable genome editing (Ministry of Science and Technology of the People’s Republic of China, 2017). However, in the Chinese context, the term “measure,” when used in the name of a law, suggests a focus on principles rather than required and detailed provisions, which might lead to implementation issues. Due to the lack of essential punishment effectiveness in these measures, as well as professional supervision or ethical standards, there is no legal basis to assume obligation for technological damage compensation and dispute resolution procedures in the aftermath of situations like the “He Jiankui Affair” (Greely, 2019).

Because of the aforementioned factors, China must accelerate the process of updating its legal and regulatory framework, resolve the interface between professional measures, ethical principles, and punitive laws, and address the lack of a basic law of biosafety that reconciles and coordinates relevant biotechnology management regulations and measures on pathogenic microorganisms, clinical research, human genetic engineering (see below Table 1).

**TABLE 1:** Existing laws, regulations, and measures related to medical biotechnology legislation in China issued from the 1980s to 2018 (China National Center for Biotechnology Development, 2019).

General
Measures on the safety in biotechnology research and development (Ministry of Science and Technology, 2017)
Human genetic engineering
Measures on the management of genetic engineering safety (State Science and Technology Commission, 1993)
Quality control points for clinical researches of somatic human genome therapy (Ministry of Health, 1993)
Measure on human assisted reproductive technology (Ministry of Health, 2001)
Specifications for human assisted reproduction technology (Ministry of Health, 2003)
Ethics
Ethical principles guiding human embryonic stem cell research (Ministry of Science and Technology and Ministry of Health, 2003)
Guidance on the ethical treatment of experiential animals (Ministry of Science and Technology, 2006)
Ethical review methods for human biomedical research (National Health and Family Planning Commission, 2016)
Ethical principles for human assisted reproductive technology and human sperm bank (Ministry of Health, 2003)
Export control of dual-use goods and technologies
Regulations on the export control of dual-use biological products and related equipment and technologies (State Council, 2002)
Measures for the management of import and export licenses of dual-use items and technologies (Ministry of Commerce and General Administration of Customs, 2005)
Measures for the management of general licenses for the export of dual-use items and technologies (Ministry of Commerce, 2009)
Pathogenic microorganisms
Measures on the preservation of medical microorganisms in China (Ministry of Health, 1985)
Regulations on the administration of deposit of microorganisms in China (State Science and Technology Commission, 1986)
Regulations on the management of pathogenic microorganism laboratories biosafety (2004; amendments in 2016 and 2018)
Categorized directory of animal pathogenic microorganisms (Ministry of Agriculture, 2005)
List of human pathogenic microorganisms (Ministry of Health, 2006)
Regulations on the management of the highly pathogenic bacteria, viruses or samples transportation (Ministry of Health, 2006)
Measures on the depository management of the animal pathogenic bacteria, viruses (Ministry of Agriculture, 2008)
Measures for the depository institution of species of human-infecting pathogenic microorganisms (Ministry of Health, 2009)
Measures on the management of microbial agents for import and export in environmental protection (Ministry of Environmental Protection and General Administration of Quality Supervision, Inspection, and Quarantine, 2010)

## The Accelerated Legislation and Legal Governance on Medical Biotechnology in the Second Round in China

Recent advancements in genetic engineering are having a significant impact on China’s medical biotechnology legislation. In the last 3 years, the administration has launched a new wave of legislative initiatives (See below Table 2).

**TABLE 2:** China’s medical biotechnology legislation enacted from 2018 to the present.

General
Civil Code of the People’s Republic of China, Article 1009 (the Third Session of the Thirteenth National People’s Congress, 2020) (Civil Code of the People’s Republic of China, 2020)
The amendment (XI) to the criminal law of the People’s Republic of China, article 331,336B (the 24th Session of the Standing Committee of the 13th National People’s Congress, 2021) (Amendment (XI) to the Criminal Law of the People’s Republic of China, 2020)
Biosecurity law (NPC Standing Committee, 2021) (Biosecurity Law of the People’s Republic of China, 2020)
Regulations on safety management of biotechnology research and development (Ministry of Science and Technology, coming soon) (Ministry of Science and Technology of the People’s Republic of China, 2019)
Clinical Research
Regulations on clinical application of new biomedical technologies (Ministry of Health, coming soon) (Ministry of Medical and Health of the People’s Republic of China, 2019)
Interim measures on the management of human biological samples for scientific research in medical and health institutions (Ministry of Health, coming soon) (Ministry of Medical and Health of the People’s Republic of China, 2022)
Ethics
Opinions to strengthen governance over ethics in science and technology (the State Council, 2022) (State Council of the People’s Republic of China, 2022)

In December 2019, a Chinese court in Shenzhen found He Jiankui and two others guilty of breaching Article 336 of the Criminal Law of the People’s Republic of China, which forbids engaging in medical activities without a license (Xinhua News, 2019a). Although He Jiankui’s conviction is Article 336 is on a legal basis, the Chinese biotechnology legislative framework has apparent flaws, particularly in the related measures and guidelines, which can be rendered inefficient or inadequate in practice and must be addressed.

On 28 May 2020, the Civil Code of the People’s Republic of China was adopted at the Thirteenth National People’s Congress’s Third Session. Article 1009 specifies that medical and scientific research involving human DNA, embryos, or the like must be carried out in conformity with applicable laws, administrative regulations, and state regulations and must not jeopardize human health, offend ethics and morals, or harm the public interest (Civil Code of the People’s Republic of China, 2020). This is the first time in China that medical and scientific research involving human genes and embryos has been precisely stated in a sense of a legal trial.

On 1 March 2021, the Criminal Law Amendment (XI), which was enacted during the 24th Session of the Standing Committee of the Thirteenth National People’s Congress, went into effect. “Whoever implants any genetically edited or cloned human embryo into the body of a human being or animal, or implants any genetically edited or cloned animal embryo into the body of a human being shall, if the circumstances are serious, be sentenced to imprisonment of not more than 3 years or limited incarceration and a fine, or be sentenced to a fine only; or if the circumstances are particularly serious, be sentenced to imprisonment of not more than 3 years or limited nor more than 7 years and a fine.” (Amendment (XI) to the Criminal Law of the People’s Republic of China, 2020) China has implemented stringent rules, blanket prohibitions, or moratoria that prohibit the implantation of any genetically altered or cloned human embryo into the body of a human being or animal, regardless of the reason.

In addition, on 15 April 2021, the Chinese Biosecurity Law went into effect. It is crucial in guaranteeing the healthy growth of biotechnology because it is a fundamental, comprehensive, systematic, and encompassing law that fills the void left by China’s lack of a basic law on biosafety and biosecurity (Biosecurity Law of the People’s Republic of China, 2020). The Biosecurity Law prohibits biotechnology research, development, and application activities that endanger national biosecurity, such as harming public health, degrading biological resources, and destroying ecosystems and biodiversity. It also outlines the scope of biotechnology-related biosafety and biosecurity, to promote and protect biotechnology development while prohibiting and restricting the use of biological agents or biotechnology to harm national security. China is attempting to enhance its medical biotechnology legislation, which encompasses themes such as genetic engineering, and clinical research, to attain these aims.

Meanwhile, the Chinese President has recently asked the Ministries to make recommendations on how to assess social risks and ethical challenges, as well as how the developing field of related sciences and technologies can maximize public benefits, minimize risks, advance relevant laws and regulations, and adhere to appropriate ethical boundaries (Xinhua News, 2019b). This would further accelerate the medical biotechnology legislation. Although they are not specifically based on a provision governing relevant biosafety in biotechnology, the Scientific and Technological Advanced Law, which was amended recently, and the Opinions to Strengthen Governance over Ethics in Science and Technology were published by the State Council, with the former stating that “Actions that endanger human health or violate scientific and technological ethics will be registered in a Chinese government database of significant scientific research integrity violations” (Amendment (II) to the Scientific and Technological Advanced Law of the People’s Republic of China, 2021). The latter shows that the violations of science and technology ethics will be thoroughly investigated and dealt with by the Chinese government (The State Council of the People’s Republic of China, 2022).

**FIGURE 1:**
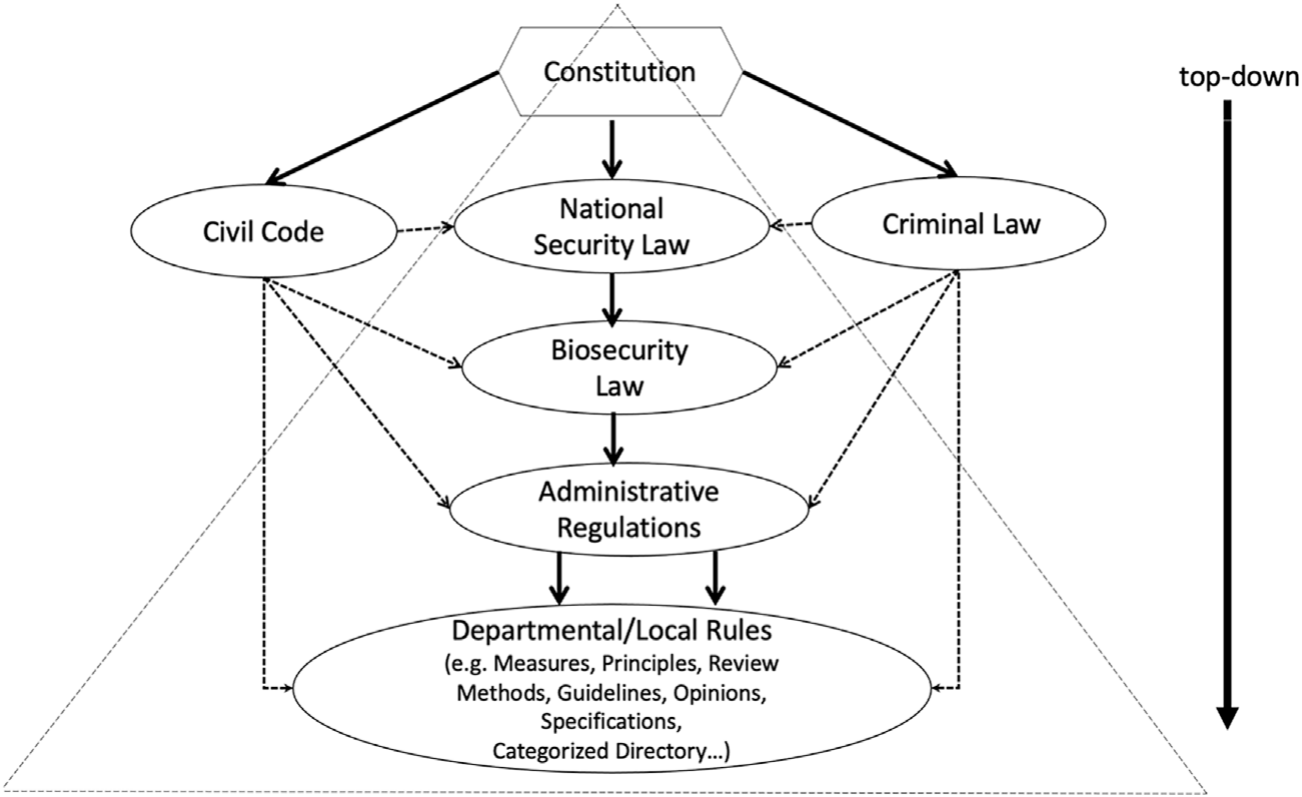
Chinese legal framework in biosafety and biosecurity.

In general, China is attempting to integrate a patchwork of existing regulations and measures relating to the emerging field of medical biotechnology into a comprehensive legal framework: the constitution oversees “national security law,” which covers a wide variety of issues related to national security, including biosafety and biosecurity. Biotechnology legislation has been incorporated into several competent administrative ministries’ administrative rules for specific implementation as an important aspect of regulation in the eight basic categories of the Biosecurity Law released in 2021. The ministries and local governments are also guided by these administrative regulations in developing applicable departmental rules, such as guidelines, measures, and principles, as well as local rules. Meanwhile, China is attempting to use the Civil Code and Criminal Law to explicitly identify actions connected to medical biotechnology safety and security in legal trials, to address the lack of a legal basis for assumed obligations for technological damages and dispute resolution procedures (See below Figure 1).

## A Close Review of the Biosecurity Law of PRC Relating to Medical Biotechnology Legislation

As an important part of regulation in the eight primary categories of the Biosecurity Law published in the year 2021, biotechnology legislation has been developed to meet the domestic goals of reducing risks to biotechnology research, development, and implementation (China Daily News, 2022b). It lays out the strategic goals of integrating biotechnology-related biosafety and biosecurity threats into the “People-Centered” philosophy of development (China Daily News, 2021), as well as promoting ecological civilization, which is based on the idea that economic and technological progress should not be at the expense of resource depletion and environmental degradation (China Daily News, 2022c). According to the Biosecurity Law, maintaining stable and healthy biotechnology development is a crucial component of a country’s ability to avoid and respond to biological agents and related dangers effectively. It also defines effective biotechnology governance as forbidding biotechnology research, development, and application activities that endanger public health, harm biological resources, destroy ecosystems and biodiversity, or pose other biosafety or biosecurity risks, as well as acting ethically. To fulfill these objectives, it has eight articles in a special chapter stipulating regulations of the components of China’s oversight system as it relates to the mandates of the research institutions: classification management; approval regime and filing system; traceability management; ethical review of clinical research; and risk assessment mechanism.

At this moment, the Biosecurity Law does not believe that it is necessary to create additional agencies or oversight organizations dedicated solely to biotechnology. Rather, it specifies that the National Health Commission (NHC), the Ministry of Science and Technology (MST), and the Ministry of Agriculture and Rural Affairs (MARA), all of which are part of the State Council, have substantial supervision responsibilities for biotechnology. These authorities have jurisdiction over everything from the lab to the field, the environment, the workplace, and the market. Clarity, collaboration, and responsibility throughout the government are required to ensure responsible stewardship in the realm of biotechnology. The law also calls for the creation of a National Biosafety/Biosecurity Coordination Mechanism (NBCM), which would bring together multiple ministries under the State Council to keep the government up to date on new advances, risks, and opportunities as the sector develops. These actions will be carried out under the Office of the National Biosafety/Biosecurity Coordination Mechanism (ONBCM), which is subordinate to the NHC. The NHC oversees biotechnology regulation in cooperation with relevant ministries, while the ONBCM is in charge of day-to-day operations and national monitoring. Meanwhile, effective biotechnology monitoring is based on the three ministries’ classifications of activities and agents, as well as an assessment of the dangers posed by the process used to operate them. Furthermore, the regulation has ramifications beyond China’s borders. For example, according to the Biosecurity Law, companies and individuals that do not have the legal status of Chinese legal persons are prohibited from engaging in high/medium risk biological research and development activities in China. It means that foreign investors who do not have the status of a Chinese legal person should not engage in related activities, according to the Company Law of the People’s Republic of China.

One of the notable features of the Biosecurity Law is its attention devoted to calling for prudent vigilance, establishing processes for assessing likely benefits along with assessing safety and security risks both before and after projects are undertaken. In general, China is adhering to the precautionary principle as it believes that uncertainty in science and technology should not become an excuse for the delay in adopting measures to prevent harm or threats, arguing that whoever carries on the development of biotechnology is obliged to bear the burden of proof of no harm. Should any biotechnological achievement carry risks on which no scientific consensus has been gained, there would be a need to impose oversight for prevention and precaution. The precautionary principle arose mostly from European debates and resolutions on environmental issues and genetically modified foods (Gollier et al., 2000). It is critical for reducing the risk that scientific research, products, or facilities will have unforeseen consequences that endanger populations. One of the premises is that if there is a societal obligation to safeguard the public or the environment, that responsibility should be reduced only when research shows that harm is unlikely to occur (Forge, 2010). These recommendations do entirely concur with the related biotechnology part of the Biosecurity Law, which focuses on protecting people’s lives and health, promoting, and safeguarding the development of biotechnology, and prohibiting the use of biological agents or biotechnology to harm national security.

The law is trying to pull together the wide array of existing regulations and measures from a loose collection applied to the emerging field of medical biotechnology into a fully integrated legislative system. However, there is still room for improvement for it to rise to the mounting challenges.

First, there is a predicament in which it is difficult to maintain a delicate balance between the regulations’ specificity and excessive generality. Regarding the research and commercialization of biotechnology, the State Council’s regulations and ministerial measures put too much emphasis on principles but do not always clearly define the liability and punishment for violations. Likewise, it also needs to deal with the relationship between both the State Council regulations and ministerial measures. According to the characteristics of the Chinese legal system, the administrative laws only address administrative responsibilities for violations, such as the Biosecurity Law’s focus on specifying monetary penalties and the administrative responsibility of institutions. The Civil Code and the Criminal Law contain special provisions involving criminal and civil responsibilities for violation. In light of the time consumed in the amendment of them both, lawmaking is often lagging evidenced broadly such as in the “He Jiankui Affair.” Furthermore, while China has adopted a Criminal Law prohibiting the implantation of any genetically altered or cloned human embryo into the body of a human being or animal, its application to specific sentences has remained overly broad due to the lack of a definition for “severe or extremely serious conditions.”

Second, a dilemma exists between the demand for cooperation and the distribution of authority and responsibility among multiple government departments and agencies. The chief of the NBCM’s Office was a lower-ranking official in an average ministry among many equals, without sufficient authority to bring together all parts of the government with a stake in biotechnology governance. Each ministry would bring its interests to the NBCM, which would face challenges in formulating and implementing regulations and measures. As a result, the NBCM’s operating mechanism would fall apart as efforts from participating ministries were not properly coordinated, potentially resulting in gaps, conflicts, and inconsistencies in the legislation (Cao, 2021). Similarly, the MARA is responsible for regulating the research, development, and application of biotechnology in crops, the NHC is responsible for regulating the research, development, and application of medicine and clinical, and the MST is primarily responsible for regulating basic research and industrial development in life sciences, according to existing ministerial regulations. Biotechnology, on the other hand, creates biosafety and biosecurity concerns that are far from idealized to be easily characterized by the product’s application. Because of the absence of clarity and accountability in the division of work, the content of laws established by several ministries is likely to overlap and even clash. It is therefore essential that an office administration with sufficient authority not only brings together all parts of the government with a stake in biotechnology but also leads an interagency process to identify and clarify, existing oversight authorities.

Third, while having fairly comprehensive and systematic coverage, the medical biotechnology legislation in China is short of non-traditional scientists’ provisions, which has been a long-existing problem. Especially in terms of technological development trends, it also poses some unusual potential risks, as “hobbyist” or “do-it-yourself” (DIY) scientists and others outside of traditional institutions conduct biological research in unknown places. There is potential for misuse, however—though, at this time, misuse is largely related to self-harm (Lee, 2017; Zayner, 2018). Groups such as DIYbio are loosely organized networks of self-described “citizen scientists” coming together with a common interest in the tools, methods, and applications, rather than shared professional affiliations or policy responsibilities (Mullin, 2018). Due to the lack of regulatory effectiveness in the traditional laws and regulations targeted at these communities, organized efforts to engage this group in discussions on safety and security and to foster a commitment to responsible stewardship will be increasingly important. These risks must be identified and anticipated with regulations and policies to assess and respond while calling for different legislation is also warranted.

Fourth, a lack of effective regulatory restraint on emerging biotechnologies such as synthetic biology not only exposes the conspicuous limitations of China’s existing biotechnology regulatory regime but also poses great challenges to global biosafety and biosecurity governance. Despite the Chinese government’s efforts to limit the hazards connected with biotechnology, accidents involving biosafety and biosecurity are inevitable. Despite the Chinese government’s efforts to restrict the risks associated with biotechnology, biosafety, and biosecurity incidents are unavoidable (China Daily News, 2022d). At present, China’s oversight of biotechnology relies on the assessment of the risks posed by the materials, products, and processes used to generate them. However, one of the biggest challenges in the oversight of emerging biotechnologies is their capacity to create novel entities that are increasingly dissimilar to known agents or organisms, making potential risks harder to assess. For example, the ability to use synthetic biology tools to produce new variants and new traits may yield many different organisms than would likely be developed through natural selection or traditional bioengineering techniques, affecting safety (Gronvall, 2018). Therefore, calls for different legislation are also warranted. In addition, gene and oligonucleotide sequences or parts can be commercially obtained with ease, and reagents and automated equipment for synthesizing nucleic acid sequences are available as well (Presidential Commission for the Study of Bioethical Issues, 2010). The increasing ease of access to the materials and supplies used to generate synthetic agents poses another unique oversight challenge for China’s “materials-products generated-process used to generate” regulatory framework.

Furthermore, consideration of medical biotechnology regulation in China has thus far focused on efforts to balance the risks and potential benefits of research and development activities. Although the precautionary principle is a statutory requirement under the Biosecurity Law in China, the field of medical biotechnology legislation can proceed responsibly by embracing neither the precautionary principle nor the innovation principle, which allows science and technology to advance uninhibitedly. According to the Regulations on Safety Management of Biotechnology Research and Development (Draft), China appears to propose a balanced dynamic monitoring system, early warning, and assessment that carefully monitors, identifies, and mitigates potential and realized harms over time, which could be a particularly valuable way to prepare for the emergence of unexpected risks that require rapid identification and creative responses in the future.

Finally, in biological sciences, the autonomy of relevant stakeholders is a crucial element (Tucker and Tucker, 2012). Current biosecurity threats are essentially related to human misuse and abuse and originate from the out-of-control conduct of relevant individuals in the face of many internal and external factors. Because biologists are the first line of defense against biotechnology misuse, their biosecurity awareness, and moral self-discipline are essential for prevention. The solution to preventing biosafety and biosecurity threats has been considered to be primarily through legal and self-governance mechanisms. Professional standards, codes of ethics, education, and awareness initiatives are all part of the latter (Xue et al., 2021). The Biosecurity Law and relative regulations appear to be under-investing in the self-governance mechanisms, particularly in terms of implementation. Although scientists from China and the United States collaborated to produce the Tianjin Biosecurity Guidelines for Codes of Conduct for Scientists to promote a culture of responsibility and guard against such misuse (United Nations Digital Library, 2021), the most efficient approach for China’s policymakers to prepare for prohibitive biotechnology policies is through laws, which should be enforced by legislation and affect regulation, measures or guidelines, as well as the articulation of relevant codes of conduct, etc.

## Discussion and Conclusion

In this paper, we discuss and evaluate China’s legislative development in medical biotechnology legislation, as well as the backdrop in which China has endeavored to handle both the need for growing internal technology and the changing external environment. The key to attaining these objectives is to accelerate the process of changing its legal and regulatory framework regularly. After the “He Jiankui affair,” Chinese policymakers have incorporated medical biotechnology-related biosafety and biosecurity into the national strategic goals of a “People-Centered” approach to develop and promote an ecological civilization. Meanwhile, China is attempting to integrate a patchwork of existing regulations and measures relating to the emerging field of medical biotechnology into a comprehensive legal framework and has begun to address the lack of a legal basis for assuming responsibility for technological damages and dispute resolution procedures, rather than relying on a large number of administrative regulations without actual punitive effect. In general, China follows the “precautionary principle” because it thinks that uncertainty in science and technology should not be used to justify delaying the adoption of measures to prevent injuries or dangers, stating that whoever advances biotechnology must face the burden of proof of no harm. There would be a need to impose oversight for prevention and precaution if any biotechnology breakthrough that carries risks on which no scientific consensus has been reached. Therefore, China aims to implement a hybrid governance model of administrative regulations and laws with punitive repercussions due to the signaling effects of administrative regulations, which might be made inadequate in practice. A similar model has been adopted by Argentina (Argentina Senate, 2019), Brazil (Senate, 2005), and other Latin American countries. This approach is similar to the EU’s “safe enough” framework for technologies. The “safe enough” narrative asserts that achieving a certain level of safety is sufficient for a technology to be rolled out unhindered. However, it is worth noting that the EU has begun to look into the shortcomings of the “safe enough” framework, such as the question of “how safe is safe enough” and the limitations of restricting ethical and governance concerns to safety considerations (European Group on Ethics in Science and New Technologies, 2021).

At the same time, China follows the “precautionary principle” which differs from the United States’ medical biotechnology regulatory method. The United States argues that it will not issue policies for tighter monitoring of sophisticated biotechnology until biosafety and biosecurity hazards have been established, claiming that intellectual freedom for invention is also important, even though biosafety risks must be anticipated (National Human Genome Research Institute, 2013). Although different countries have taken different regulatory paths, the principal goal of effective medical biotechnology regulatory policy, which includes “top-down” setting of general objectives by the active political leadership and “bottom-up” innovation in the application, is the elimination of risk or willingness to take an acceptable risk based on the value of the opportunity (Raybould, 2021). China is seeking to call for prudent vigilance, building processes for assessing anticipated benefits as well as assessing safety and security threats both before and after initiatives are performed, to achieve a reasonable balance between precaution and innovation. Meanwhile, China’s approach to addressing medical biotechnology risks and problems, as well as its adoption of a hybrid legislative model of administrative regulations and laws with penal consequences, are representative of other countries and have ramifications for them. Regular and periodical review of the relevant evidence from fast-developing biotechnology and any cases of law-breaking is the key to addressing risk assessment and risk management and striving for a proportionate balance between safety/precaution and innovation so that to ensure the best application of this “precautionary principle.” Given medical biotechnology’s rapid advancement, it is necessary for countries around the world to examine the governance situation around biosafety and biosecurity and to expeditiously consider options for establishing a comprehensive, credible, and long-lasting regulatory framework. In particular, this understanding would serve as a link between scientific communities and national or international governance entities. We argue that the purpose of biosafety and biosecurity governance needs to reflect on and institutionalize policies in grassroots practice according to local realities. Experiences gathered from China’s governance would aid in the development of a scalable road map for the future.

## Data Availability

The original contributions presented in the study are included in the article/supplementary material, further inquiries can be directed to the corresponding author.
